# High flow nasal cannula for acute respiratory failure due to COVID‐19 in patients with a ‘do‐not‐intubate’ order: A survival analysis

**DOI:** 10.1111/crj.13573

**Published:** 2022-12-30

**Authors:** Illaa Smesseim, Kirsten Mooij‐Kalverda, Lisa Hessels, Daniel A. Korevaar, Burak Atasever, Hjalmar de Graaff, Abraham Goorhuis, Esther Nossent, Lieuwe Bos, Peter Bonta, the Amsterdam UMC HFNC study group:, Joost van den Aardweg, Wim Boersma, Ivo van der Lee, Herre J. Reesink

**Affiliations:** ^1^ Department of Pulmonary Medicine, Amsterdam UMC Free University Amsterdam and University of Amsterdam Amsterdam The Netherlands; ^2^ Department of Pulmonary Diseases Noordwest Ziekenhuisgroep Alkmaar The Netherlands; ^3^ Department of Pulmonary Diseases Spaarne Gasthuis Haarlem The Netherlands; ^4^ Department of Pulmonary Diseases Onze Lieve Vrouwe Gasthuis Amsterdam The Netherlands; ^5^ Department of Infectious Diseases Amsterdam University Medical Center Amsterdam The Netherlands; ^6^ Amsterdam Cardiovascular Sciences Research Institute, Amsterdam UMC Amsterdam The Netherlands; ^7^ Intensive Care Amsterdam University Medical Centers—location AMC Amsterdam The Netherlands

**Keywords:** ARDS, covid‐19, dni‐order, do‐not‐intubate order, hfnc

## Abstract

**Introduction:**

High flow nasal cannula (HFNC) reduces the need for intubation in patients with hypoxaemic acute respiratory failure (ARF), but its added value in patients with severe coronavirus disease 2019 (COVID‐19) and a do‐not‐intubate (DNI) order is unknown. We aimed to assess (variables associated with) survival in these patients.

**Materials and methods:**

We described a multicentre retrospective observational cohort study in five hospitals in the Netherlands and assessed the survival in COVID‐19 patients with severe acute respiratory failure and a DNI order who were treated with high flow nasal cannula. We also studied variables associated with survival.

**Results and discussion:**

One‐third of patients survived after 30 days. Survival was 43.9% in the subgroup of patients with a good WHO performance status and only 16.1% in patients with a poor WHO performance status. Patients who were admitted to the hospital for a longer period prior to HFNC initiation were less likely to survive. HFNC resulted in an increase in ROX values, reflective of improved oxygenation and/or decreased respiratory rate.

**Conclusion:**

Our data suggest that a trial of HFNC could be considered to increase chances of survival in patients with ARF due to COVID‐19 pneumonitis and a DNI order, especially in those with a good WHO performance status.

## Graphical abstract

High flow nasal cannula (HFNC) reduces the need for intubation in patients with hypoxaemic acute respiratory failure (ARF). Our data suggest that a trial of HFNC could be considered to increase chances of survival in patients with ARF due to coronavirus disease 2019 (COVID‐19) pneumonitis and a do‐not‐intubate (DNI) order, especially in those with a good WHO performance status.

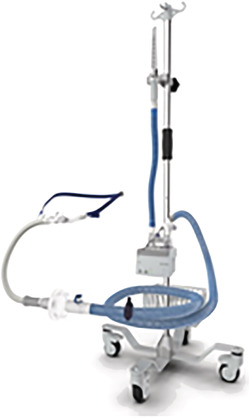

## 1 INTRODUCTION

Hypoxaemic acute respiratory failure (ARF) is one of the hallmarks of severe coronavirus disease 2019 (COVID‐19).^1^ High flow nasal cannula (HFNC) is a method to deliver high fractions of body temperature heated and humidified inspired oxygen using high flow. HFNC achieves approximately 5 cmH_2_O of positive end‐expiratory pressure (PEEP), improves oxygen saturation at the same fraction of inspired oxygen (FiO_2_) and contributes to CO_2_ washout from the anatomical dead space.^2^ HFNC can reduce the need for intubation in patients with ARF not related to COVID‐19.^3^ The World Health Organization and European Respiratory Society guidelines made a conditional recommendation in favour of a trial of HFNC in patients with COVID‐19‐related ARF before intubation.^4^ The ROX index ([peripheral oxygen saturation (SpO₂)/FiO₂] /respiratory rate) is considered a marker for HFNC success and might be used to monitor treatment effect.^5^, ^6^

So far, studies on HFNC have mostly focused on the prevention of intubation. Little is known about outcomes in patients with ARF due to COVID‐19 and a ‘do‐not‐intubate’

(DNI) order. In such patients, recent studies reported almost 97% mortality within 28 days in patients requiring 15 L/min of oxygen, in the absence of HFNC.^7^, ^8^ In this study, we aimed to assess (1) survival in patients with severe ARF due to COVID‐19 who had a DNI order and were treated with HFNC, (2) variables associated with survival and (3) associations between dynamical change in ROX index and survival.

## 2 METHODS

We conducted a multicentre retrospective observational cohort study in two university hospitals (Amsterdam University Medical Centers, location Academic Medical Center and VU Medical Center) and three large general hospitals (Noordwest Ziekenhuisgroep Alkmaar, Spaarne Gasthuis and Onze Lieve Vrouwe Gasthuis) in the Netherlands. All adult patients with a DNI order treated with HFNC (Optiflow from Fisher&Paykel) for hypoxaemic ARF due to COVID‐19 in a non‐ICU‐setting between 01 March 2020 and 31 January 2021 were included. Patients with hypercapnia or serious comorbidities with limited life expectancy were considered ineligible. COVID‐19 pneumonitis was confirmed by PCR for SARS‐CoV‐2 and compatible chest imaging. Severe hypoxaemic ARF was defined as a SpO_2_ ≤ 95% despite 15 L/min of oxygen through a nonrebreathing mask.^9^ The decision to apply a DNI order and initiate HFNC in these patients was at the discretion of the treating medical team. Factors considered in the decision‐making process were performance status, comorbidities, quality of life, cognition, age, prognosis and wishes of the patient or his/her legal representative. The study protocol was exempted from ethical approval by our institute's medical ethics committee, as the Medical Research Involving Human Subjects Act does not apply to this retrospective observational study. [Correction added on 10 January 2023, after first online publication: ‘Academic Medical Centre’ has been updated to ‘Academic Medical Center’ in the first sentence of this paragraph.]

Demographic and clinical variables were recorded from patient records. The ROX index was calculated prior to initiation of HFNC and every 24 h thereafter (+/− 2 h). WHO performance status (ranging from 0 [i.e. fully active] to 4 [i.e. completely disabled]) prior to hospital admission was recorded by the treating physician.

Survival at 30 days after HFNC initiation was calculated as a proportion. We assessed associations of the following predefined variables with survival (per one unit increase for continuous variables, unless reported otherwise): age (years), gender (female [reference category] versus male), BMI (kg/m^2^), WHO performance status (2–4 [reference category] versus 0–1), D‐dimer (mg/L), hospital length of stay prior to HFNC initiation (days) and ROX index ([SpO₂/FiO₂] /respiratory rate) prior to HFNC initiation. The association between dynamic change in ROX index and mortality was evaluated using linear mixed effect model analysis using the patient as random intercept and using ROX index, days from HFNC initiation and an interaction term of the two as fixed effect and with mortality as the dependent variable. Patients with missing ROX index values (10/79) were excluded from the latter analysis, but included in the other analyses. All analyses were performed in R through the R‐studio interface.^10^

## 3 RESULTS

We enrolled 79 patients, of whom 64 (81.0%) were males, with a median age of 78 years (IQR: 72–81). The most frequent comorbidities were cardiovascular disease (*n* = 52; 65.8%) and diabetes mellitus (*n* = 31; 39.2%). During admission, most patients received corticosteroids (*n* = 72; 91.1%), while some received antibiotics (*n* = 25; 31.6%). All patients received prophylactic anticoagulation, except for those who already used therapeutic anticoagulation prior to hospitalisation (*n* = 25; 31.6%).

**FIGURE 1:**
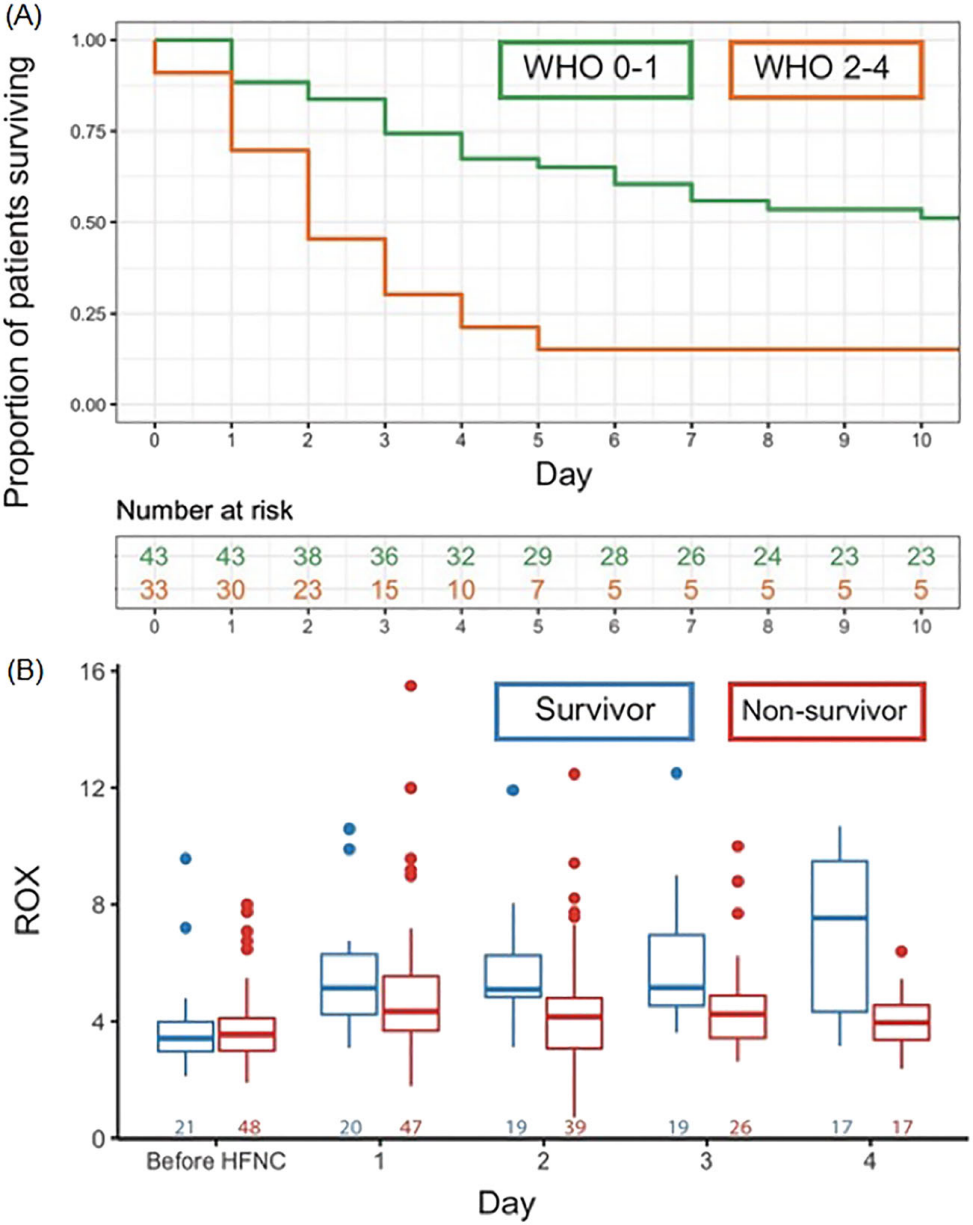
(A) Association between WHO performance status prior to high flow nasal cannula (HFNC) initiation and mortality. (B) Daily median ROX index differences between survivors and nonsurvivors

Survival at 30 days after HFNC initiation was 32% (*n* = 25) and was the same at 60 days. In univariable logistic regression analysis, WHO performance status and hospital length of stay prior to HFNC initiation were significantly associated with mortality (OR_WHOstatus0–1_: 6.65, 95%‐CI: 2.09–21.1; OR_HospitalLOS_: 2.07, 95%‐CI: 1.03–4.18) (Figure 1A). Age, gender, BMI, D‐dimer and ROX index prior to HFNC initiation were not significantly associated with mortality (OR_Age_: 1.08, 95%‐CI: 0.66–1.78; OR_GenderMale_: 0.45, 95%‐CI: 0.14–1.41; OR_BMI_: 1.04, 95%‐CI: 0.56–1.94; OR_D‐dimer_: 1.01, 95%‐CI: 0.56–1.94; OR_ROX_: 0.85, 95%‐CI: 0.57–1.27).

Linear mixed effect model analysis showed that ROX index improved with days on HFNC (β_DaysHFNC_: 0.41, 95%‐CI: 0.25–0.55) in the overall population, while average ROX values were not significantly different between survivors and nonsurvivors (β_Mortality_: 0.24, 95%‐CI: −0.99 to 1.47). The interaction term was significantly associated with ROX index (β_DaysHFNC:Mortality_: ‐0.73, 95%‐CI: −0.95 to −0.50), indicating that the slope in ROX index differed between survivors and nonsurvivors. This is illustrated in Figure 1B, where the median ROX index improves over time in survivors, but not in nonsurvivors, with the largest difference at day 4.

## 4 DISCUSSION

This is the first study reporting on outcomes in patients with severe ARF due to COVID‐19 and a DNI order being treated with HFNC. We found that one‐third of patients survived after 30 days. Survival was 43.9% in the subgroup of patients with a good WHO performance status (0–1), while it was only 16.1% in patients with a poor WHO performance status (2–4). HFNC resulted in an increase in ROX values, reflective of improved oxygenation and/or decreased respiratory rate. Patients who were admitted to hospital for a longer period prior to HFNC initiation were less likely to survive; the delay in HFNC initiation in these patients is explained by the fact that HFNC was only initiated when they met criteria for severe hypoxemic ARF at presentation or during hospital stay. As such, initiation of HFNC may have provided a longer window to benefit from medical treatments such as corticosteroids and tocilizumab in patients presenting with severe ARF, while the patients with later start of HFNC during their hospital stay had already deteriorated despite such treatments. ROX index trajectories were significantly different between survivors and nonsurvivors, which is in line with evidence on intubation risk in the non‐COVID‐19 population.^6^

Main strengths of this study are the multicentre design and the novelty of the data. Limitations are the retrospective design, lack of a matched control group of patients who were not treated with HFNC and the fact that the decision to initiate and terminate HFNC was at the discretion of the treating physician, which may have led to varying practices across and within participating centres. In addition, our study only focussed on patients with a DNI order who were considered eligible to undergo a trial of HFNC, but we did not document the number of patients that were not considered eligible nor reasons for that.

Based on these findings, we believe that a trial of HFNC could be considered in patients with ARF due to COVID‐19 and a DNI order, especially in those with a good WHO performance status (0–1). However, chances of HFNC success are limited in patients with a poor performance status and later start of HFNC during their hospital admission. Future prospective studies are needed to confirm improved survival of HFNC treatment versus other oxygen devices in this patient group.

## CONFLICT OF INTEREST

All authors have no conflicts of interest to declare that are relevant to the content of this article. All authors certify that they have no affiliations with or involvement in any organisation or entity with any financial interest or nonfinancial interest in the subject matter or materials discussed in this manuscript.

## ETHICS STATEMENT

This material is the authors' own original work, which has not been previously published elsewhere. The study protocol was exempted from ethical approval by our institute's medical ethics committee, as the Medical Research Involving Human Subjects Act does not apply to this retrospective observational study.

## AUTHOR CONTRIBUTIONS

The authors confirm contribution to the paper as follows: study conception and design: IS, PB, LB, DK and KMK; data collection: IS, LH, BA and HdG; analysis and interpretation of results: IS, PB, DK and LB; draft manuscript preparation: IS. All authors interpreted the data and critically reviewed the manuscript and approved the final version of the manuscript.

## Data Availability

The data that support the findings of this study are available from the corresponding author upon reasonable request.
